# Which surgeon demographic factors influence postoperative complication rates after total knee arthroplasty at U.S. News and World Report top-ranked orthopedic hospitals?

**DOI:** 10.1186/s42836-022-00125-8

**Published:** 2022-07-04

**Authors:** Adam M. Gordon, Andrew R. Horn, Keith B. Diamond, Mitchell K. Ng, Matthew L. Magruder, Orry Erez

**Affiliations:** Maimonides Medical Center, Department of Orthopedic Surgery, 927 49th Street, Brooklyn, New York, NY 11219 USA

**Keywords:** US News World Report, Complications, Total Knee Arthroplasty, Learning Curve, Case Volume

## Abstract

**Introduction:**

Complication rates are used to evaluate surgical quality-of-care and determine health care reimbursements. The *U.S. News & World Report* (USNWR) hospital rankings are a highly-referenced source for top hospitals. The objective of this study was to determine the surgeon demographics of those practicing at USNWR Top Ranked Orthopedic Hospitals and if any influence complication rates after total knee arthroplasty (TKA).

**Methods:**

The 2009–2013 USNWR ‘Orthopedic’ hospital rankings were identified. A database of TKA surgeons with postoperative complication rates was compiled utilizing publicly available data from the Centers for Medicare and Medicaid Services (2009–2013). Using an internet search algorithm, demographic data were collected for each surgeon and consisted of: fellowship training, years in practice, age, gender, practice setting, medical degree type, residency reputation, case volume, and geographic region of hospital. Logistic regression was used to assess the relationship between surgeon demographics and postoperative complication rates. A *P* value of < 0.008 was considered significant.

**Results:**

From 2009 to 2013, 660 orthopedic surgeons performed TKA at 80 different USNWR Top-Ranked Hospitals. Mean TKA case volume was 172 (Range, 20–1323) and age of surgeon was 50.8 (Range, 32–77). A total of 372 (56.8%) completed an orthopedic surgery fellowship. Mean adjusted 30-day complication rate was 2.24% (Range, 1.2–4.5%). After adjustment, factors associated with increased complication rates were surgeon age ≤ 42 (OR 3.15; *P* = 0.007) and lower case volume (≤ 100 cases) (OR 2.52; *P* < 0.0001). Gender, hospital geographic region, completion of a fellowship, medical degree type, and residency reputation were not significant factors.

**Discussion:**

Complication rates of total knee arthroplasty surgeons may be utilized by patients and hospitals to gauge quality of care. Certain surgeon factors may influence complication rates of surgeons performing TKA at USNWR Top Ranked Orthopedic Hospitals.

**Study Type:**

Level III, retrospective observational study.

## Introduction

Hospital rankings are a measure that allows patients to compare quality of care while influencing consumer choice of providers [1–4]. From its inception in 1990, The* U.S. News & World Report* (USNWR) hospital rankings remain the most-cited and recognizable rankings in terms of identifying top hospitals in a given specialty [5–7]. Despite widespread use by advertising agencies, hospitals, surgeons, and patients, objective measures of surgeons at these hospitals have seldom been studied for total joint arthroplasty [8]. The association between USNWR rankings and clinical outcomes following medical and surgical procedures remains conflicted, as some studies report ranked hospitals having superior results while others show similar or equivocal outcomes compared to unranked hospitals [9–16]. Since a proportion of the reputation and ranking of these hospitals is dependent on ‘expert opinion,’ it would be valuable to study the surgeon characteristics and objective postoperative outcomes of those surgeons practicing at top orthopedic hospitals [6]. As orthopedic surgery continues to evolve and stress value-based care, it is important to evaluate how sufficient the USNWR rankings are reflective of actual patient outcomes and what surgeon level factors may influence those outcomes [14, 17–19].

Complication rates, including mortality and 30-day re-admissions are used by the Centers for Medicare and Medicaid Services (CMS) as a proxy for quality of care [20]. With the implementation of bundled payment models, hospital reimbursements are tied to complications and re-admissions since these are a major contributor of potentially preventable healthcare costs [20]. Specifically, total joint arthroplasty of the hip and knee have demonstrated exponential growth in utilization and have been ongoing targets by CMS [21, 22]. Ultimately, from individual-level data (surgeons) to large healthcare networks, a better understanding of complication rates and factors influencing them is warranted. As both hospital and surgeon performance data becomes more readily available to the public, these measures may influence consumer choice and expectations of surgery [3, 23, 24].

Several single-institution and national database studies have established patient factors that influence postsurgical outcomes after total knee arthroplasty [25–28]. However, the studies evaluating surgeon level factors (years in practice, residency location, fellowship training, case volume, and others) influencing complication rates after TKA are either single-surgeon studies or derived from hospital level data and lack granularity regarding surgeon characteristics which could potentially impact the results of prior studies [29–35]. Therefore, the objective of this study was to determine surgeon demographics of those practicing at USNWR Top Ranked orthopedic hospitals and if any influence postoperative complication rates after total knee arthroplasty (TKA).

## Methods

### Data source and surgeon inclusion

A novel database reflecting TKA complication rates and surgeon demographic data was created using publicly available information. The 2009–2013 USNWR ‘Orthopedic’ hospital rankings were identified and documented [6]. We sampled all TKA surgeons rated on the ProPublica Surgeon Scorecard website who performed surgery at a USNWR Top Orthopedic Hospital during the study period (*n* = 660). To identify these TKA surgeons, the Centers for Medicare and Medicaid Services (CMS) used physician National Provider Identifiers (NPI) to link surgeons to procedures. The ProPublica Surgeon Scorecard website was used because it provides detailed individual-level data including specific surgeons who performed surgeries at the given hospitals [36]. The database reports adjusted complication rates for 16,827 surgeons operating at 3,575 hospitals for 8 surgical procedures. Only surgeons who performed more than 20 TKAs were included in the evaluation to protect patient privacy. Physicians with duplicate entries were noted as performing surgery at the given hospital with the higher hospital ranking.

### Demographic data and complications

This was a retrospective review of administrative/insurance claims from the 100% Medicare Standard Analytical Files (SAF100). Using the ProPublica Surgeon Scorecard, adjusted complication rates for elective TKA [ICD-9-CM procedure codes 81.54 (total knee replacement)] were obtained for surgeons identified in the CMS database. Complications were defined as death during initial hospital stay or hospital re-admission within 30 days for a number of possible principal diagnoses indicating a negative surgical outcome (superficial infection, deep surgical site infection, uncontrolled bleeding, malaligned orthopedic implant, peri-prosthetic fracture, acute postoperative pain, pulmonary embolism, deep venous thrombosis, sepsis and more). A panel of at least five arthroplasty surgeons reviewed principal diagnosis codes as the cause of re-admission. Reviewers indicated whether the principal diagnosis was likely to be a complication related to the index surgery and only those contributed to the adjusted complication rate. Given the complications used in this study were major and only recorded if the patient was admitted, the inter-rater reliability in judging complications was high for the panel of 5 surgeons. Any further differing judgements regarding the complications by the arthroplasty surgeons resulted in the complication being omitted from analysis. A complication such as pneumonia, which can be multifactorial and not directly an effect of the joint replacement, was not considered in our study. The panel of 5 reviewers chose to give surgeons the benefit of the doubt as this complication could be a result of anesthesia or other unknown factors. To account for confounding factors [patient age, sex, baseline health (Elixhauser comorbidity index), procedure complexity, and hospital performance] affecting a surgeon's complication rate, ProPublica implemented a generalized linear mixed effects model to compute an adjusted complication rate (ACR). This model included the following fixed effects: age, sex, and health score. This standardized all rates such that adjusted rates would reflect 30-day complications had each surgeon performed TKA surgery on a standardized group of Medicare patients in the average US hospital. More detailed information on the methodology can be found on the website [37].

After obtaining ranked hospitals and the included surgeons, demographic information for each surgeon was compiled utilizing public resources including online physician profiles and physician grading websites. All demographic data were corroborated by a minimum of 2 independent sources (www.sharecare.com, www.healthgrades.com, www.health.usnews.com/doctors, www.doximity.com). Using an internet search algorithm, demographic data for each surgeon were collected and consisted of: completing a fellowship (Yes/No), years in practice, age (years), gender, practice setting (teaching *vs.* nonteaching), medical degree type (MD *vs.* DO), residency reputation (from Doximity Residency Navigator), case volume, and geographic region of hospital.

This study was determined to be exempt from the Institutional Review Board as all data were obtained through publicly available resources and no identifiable patient information was utilized.

### Primary outcomes

The primary outcome of interest was to quantify the adjusted complication rates of TKA surgeons at USNWR Top Orthopedic Hospitals. Additionally, the objective was to understand which surgeon factors influenced short-term complication rates in the Medicare TKA population. Adjusted complication rates were evaluated as a dichotomous variable. For the purpose of this study, we evaluated surgeons above and below the mean complication rate and those > 1STD above the mean.

### Statistical analysis

Statistical analysis was performed using SPSS version 24 [International Business Machine (IBM), Armonk, NY, USA)]. Years in practice was compared as a categorical variable (≤ 10 *vs.* 10–20 *vs.* > 20 years). Age was compared between decade cohorts (≤ 40, 41–50, 51–60, 61 +) and by quartiles. Residency reputation was compared as a dichotomous variable (top 10 *vs*. > 10 and top 25 *vs*. > 25). Case volume was compared by quartiles and as a dichotomous split at the median. Multivariate Logistic regression was used to assess the effects of individual surgeon demographic factors as predictors of complication rates after TKA. For all analyses, a Bonferroni adjustment was carried out to correct for multiple comparisons with a statistical significance threshold set at *P* < 0.008.

## Results

### Surgeon and hospital characteristics

Overall, the patient cohort was derived from 1,190,631 Medicare patients who underwent TKA by 18,029 surgeons from 2009 to 2013. From 2009 to 2013, 660 orthopedic surgeons performed TKA at 80 USNWR Top-Ranked Hospitals. Surgeon and hospital characteristics are shown in Table 1. The mean TKA case volume was 172 (Range, 20–1323) and average age of surgeon was 50.8 (Range, 32–77). Three hundred seventy-two (56.8%) completed an orthopedic surgery fellowship. The mean time of years in independent practice was 18.7 years (Range, 0.1–47). The proportion of surgeons that attended a Doximity Ranked Top 10 Orthopedic surgery residency was 160/660 (24.2%). Mean adjusted 30-day complication rate for all surgeons was 2.24% (Range, 1.2–4.5%). All surgeons were employed at teaching hospitals. Further surgeon and hospital demographics are shown in Table 1.

**Table 1:** Surgeon Demographics at USNWR Hospitals

Variables	*n* (%)
**Number of Surgeons**	660
**Hospital Region**	
East	219 (33.2)
Midwest	198 (30.0)
South	109 (16.5)
West	134 (20.3)
**Teaching Hospital**	
Yes	660 (100)
No	0 (0)
**Gender**	
Female	11 (1.7)
Male	649 (98.3)
**Physician Type**	
MD	643 (97.4)
DO	17 (2.6)
**Age (Years)**^a^	50.8 (9.7)
**Age Cohorts (Years)**	
≤ 40	112 (16.9)
41–50	217 (32.9)
51–60	223 (33.8)
61 +	108 (16.4)
**Surgeon Volume**^a^	171.9 (175.5)
**Surgeon Volume (Cases)**	
≤ 50	163 (24.7)
51–100	167 (25.3)
101–229	165 (25.0)
230 +	165 (25.0)
**Residency Reputation**	
Top 10	160 (24.2)
> 10	500 (75.8)
**Fellowship Trained**	
No	288 (43.2)
Yes	372 (56.8)
**Years in Practice**^a^	18.7 (10.3)
**Years in Practice Cohorts**	
≤ 10	166 (25.2)
11–20	210 (31.8)
21 +	284 (43.0)
**Complication Rate (%)**^a^	2.24 (0.45)

USNWR = United States News & World Report

Values are Reported as N (%) except where specified

^a^Mean (STD)

### Risk factors for complication rates

**Fig. 1:**
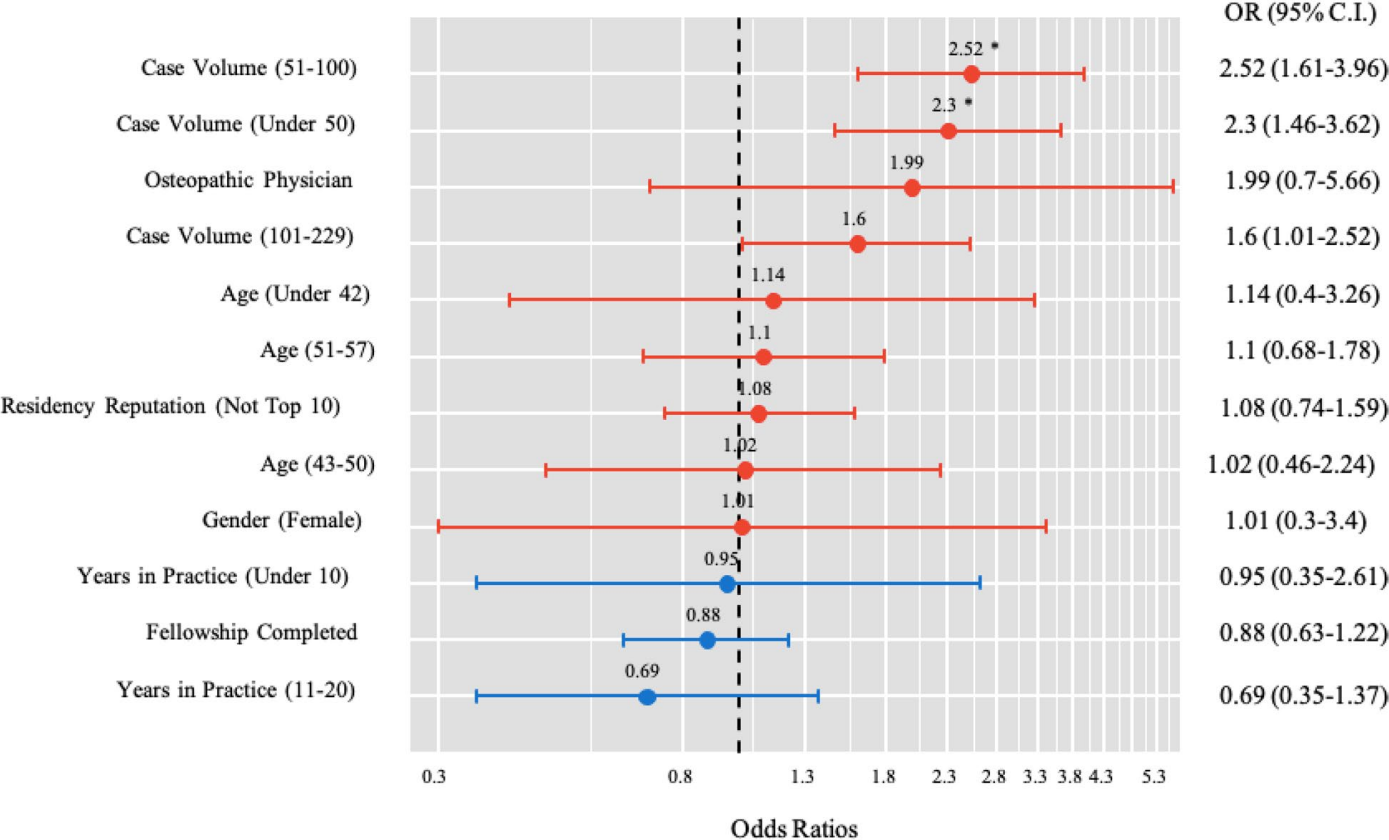
Forest Plot of Logistic Regression for Complication Rate Greater than the Mean (> 2.2%). *OR* Odds Ratio, *CI* Confidence Interval

**Fig. 2:**
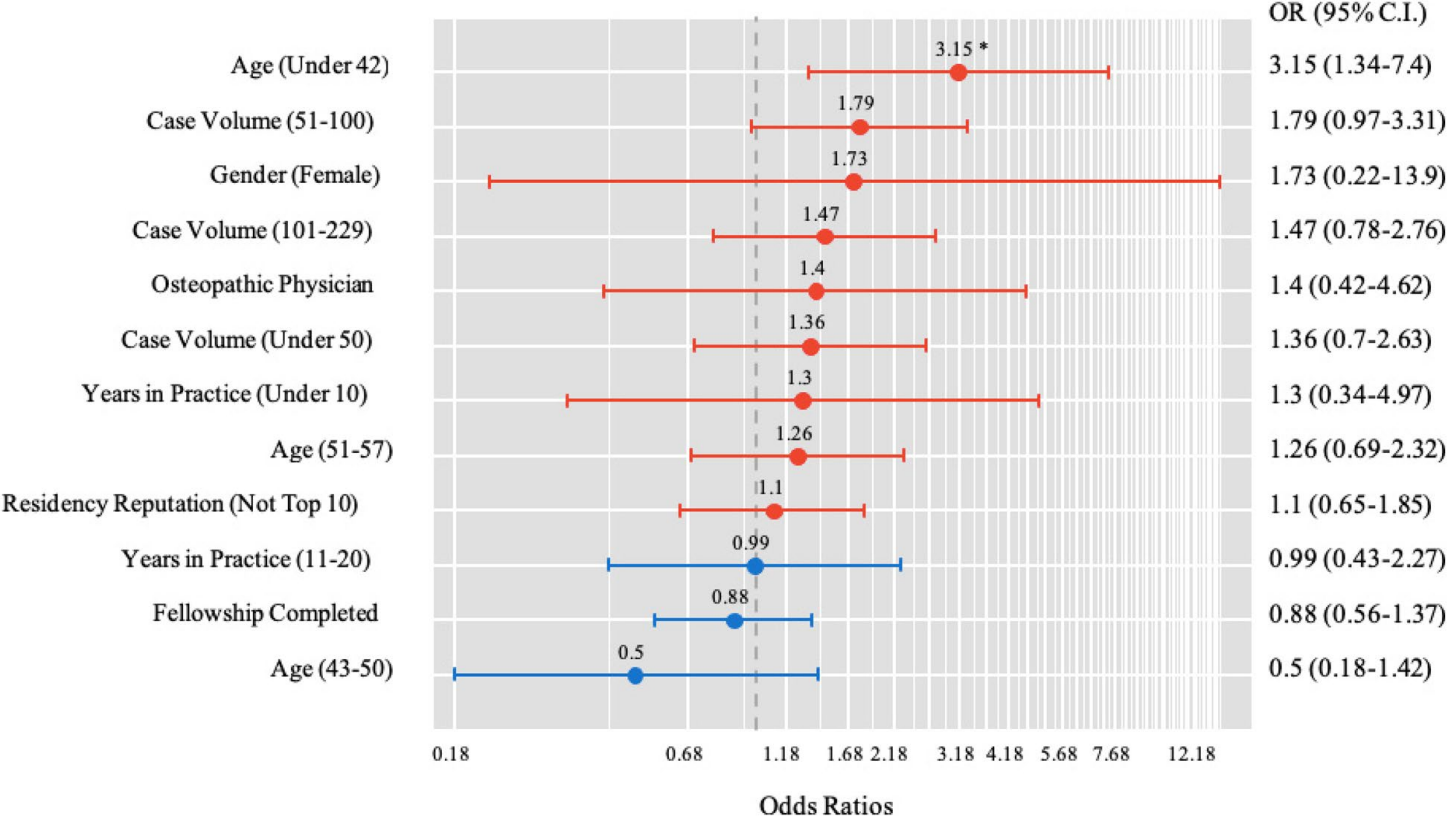
Forest Plot of Logistic Regression for Complication Rate Greater than 1STD above the Mean (≥ 2.7). *OR* Odds Ratio, *CI* Confidence Interval

Mean adjusted 30-day complication rate for all surgeons was 2.24% (STD 0.45%). Surgeon demographics for those above and below the mean are shown in Table 2. Surgeons above the mean complication rate performed significantly less cases (< 100) (*P* < 0.001). Surgeon characteristics with the highest complication rates are shown in Table 3. After adjusting for surgeon and hospital characteristics, independent factors associated with increased adjusted complication rates included surgeon age ≤ 42 years (OR 3.15, CI 1.34–7.40; *P* = 0.007) and lower case volume (≤ 100 cases) (OR 2.52, CI 1.61–3.96; *P* < 0.0001) (Fig. 1). Gender, hospital geographic region, completion of a fellowship, medical degree type, and residency reputation were all non-significant contributors (Fig. 2).

**Table 2:** Surgeon Demographics for Complication Rate above Mean

Variable	Complication Rate				*P* Value^a^
	≤ 2.2%		> 2.2%		
	*n*	%	*n*	%	
**Number of Surgeons**	365		295		
**Hospital Region**					
East	127	34.8	92	31.2	0.607
Midwest	105	28.8	93	31.5	
South	63	17.3	46	15.6	
West	70	19.2	64	21.7	
**Teaching Hospital**					
Yes	365	100.0	295	100.0	-
No	0	0.0	0	0.0	
**Gender**					
Female	6	1.6	5	1.7	0.959
Male	359	98.4	290	98.3	
**Physician Type**					
MD	359	98.4	284	96.3	0.093
DO	6	1.6	11	3.7	
**Age Cohorts (Years)**					
≤ 42	81	22.2	76	25.8	0.263
43–50	106	29.0	66	22.4	
51–57	86	23.6	73	24.7	
≥ 58	92	25.2	80	27.1	
**Surgeon Volume (Cases)**					
≤ 50	80	21.9	83	28.1	** < 0.001**
51–100	77	21.1	90	30.5	
101–229	95	26.0	70	23.7	
230 +	113	31.0	52	17.6	
**Residency Reputation**					
Top 10	95	26.0	65	22.0	0.234
> 10	270	74.0	230	78.0	
**Fellowship Trained**					
No	150	41.1	138	46.8	0.143
Yes	215	58.9	157	53.2	
**Years in Practice Cohorts**					
≤ 10	86	23.6	80	27.1	0.094
11–20	129	35.3	81	27.5	
21 +	150	41.1	134	45.4	

^a^Pearson Chi Square Test

**Table 3:** Surgeon Demographics for Elevated Complication Rate (> 1 STD above Mean)

Variable	Complication Rate				*P* Value^a^
	< 2.7%		≥ 2.7%		
	*n*	%	*n*	%	
**Number of Surgeons**	554		106		
**Hospital Region**					
East	182	32.9	37	34.9	0.696
Midwest	164	29.6	34	32.1	
South	91	16.4	18	17.0	
West	117	21.1	17	16.0	
**Teaching Hospital**					
Yes	554	100.0	106	100.0	-
No	0	0.0	0	0.0	
**Gender**					
Female	10	1.8	1	0.9	0.704
Male	544	98.2	105	99.1	
**Physician Type**					
MD	541	97.7	102	96.2	0.498
DO	13	2.3	4	3.8	
**Age Cohorts (Years)**					
≤ 42	129	23.3	28	26.4	0.031
43–50	156	28.2	16	15.1	
51–57	126	22.7	33	31.1	
≥ 58	143	25.8	29	27.4	
**Surgeon Volume (Cases)**					
≤ 50	138	24.9	25	23.6	0.234
51–100	133	24.0	34	32.1	
101–229	138	24.9	27	25.5	
230 +	145	26.2	20	18.9	
**Residency Reputation**					
Top 10	136	24.5	24	22.6	0.675
> 10	418	75.5	82	77.4	
**Fellowship Trained**					
No	239	43.1	49	46.2	0.557
Yes	315	56.9	57	53.8	
**Years in Practice Cohorts**					
≤ 10	137	24.7	29	27.4	0.135
11–20	185	33.4	25	23.6	
21 +	232	41.9	52	49.1	

^a^Pearson Chi Square Test

## Discussion

Using a nationwide Medicare sample undergoing elective TKA, we evaluated the characteristics and complication rates of surgeons at USNWR top-ranked orthopedic surgery hospitals from 2009 to 2013. Across the nation, the mean adjusted complication rate for surgeons at these facilities was 2.24%, with specific surgeon factors (younger age and lower case volume) influencing greater postoperative complication rates. Because rankings are often used by consumers, payers, and providers to direct patient care and generate popularity between facilities, understanding the association of these rankings with objective clinical outcomes is necessary to understand their association with quality care. The results of the current study may serve as a benchmark for surgeons currently practicing at these facilities or even spark further study comparing surgeons at ranked *vs.* unranked facilities. Also, the findings of our study may serve to improve patients’ perceptions of the utility of easily accessible online ranking systems.

USNWR top-ranked hospitals are typically regarded as the premier facilities to receive specialized care, however, the association of rankings and outcomes in orthopedics are both questionable and sparse. The most relevant study was done by Cram and colleagues at the hospital level, comparing primary TKA in top-ranked and non-top-ranked orthopedic hospitals [8]. They found that complications, re-admissions and hospital length of stay were similar for Medicare patients between hospital settings. Rankings are somewhat arbitrary and can mislead patients as one study compared 5 different ranking systems in orthopedics and found no hospital was ranked as ‘high-performing’ by all five rating systems [19]. To better understand potential factors that may influence the quality of care and reputation, we studied the surgeon characteristics of those performing TKA at these facilities which addresses a gap in the existing literature. Approximately 2/3 of the top-ranked orthopedic hospital TKA surgeons practiced in the Eastern and Midwestern United States. All surgeons were affiliated with a teaching institution, either at the resident or fellow level. Nearly 50% of the surgeons were 50 years of age or younger with 25% of the surgeon sample completing an orthopedic surgery residency at a top 10 ranked program. Despite the increased propensity for new orthopedic surgery graduates to complete a fellowship, we report that a little over half are fellowship-trained. Based on the results of our study, we did not find any noticeable characteristics that define TKA surgeons at USNWR top-ranked orthopedic hospitals. Further study with contemporary analysis would be helpful.

A better understanding of complication rates which are used by the Centers for Medicare and Medicaid Services (CMS) as a proxy for quality care is warranted. Risk factors associated with increased adjusted complication rates in the present study included younger surgeon age (≤ 42) and lower case volume (≤ 100 cases). These results are intuitive as the learning curve (case volume) of TKA surgeons during independent practice has been shown to be around 100 cases [30, 31]. While the lower case volume results reflect the study group as a whole, it is possible that some low-volume surgeons might have lower complication rates compared to higher volume surgeons. Historically, gender, medical degree type and residency reputation intuitively may have been used to define quality of care by the general public. As the first study to evaluate these variables in this surgeon cohort, we showed that gender, hospital geographic region, completion of a fellowship, medical degree type, and residency reputation all were non-significant contributors toward complication rate after TKA. Further longitudinal study capturing measures of patient-reported outcomes is needed to make stronger conclusions about which surgeon factors influence outcomes after TKA.

The rise in consumer-centric health insurance plans has increased the importance of patient decision-making regarding providers and hospitals. With patients playing a larger role in controlling their healthcare, understanding the factors influencing selection of specialist providers has been an area of ongoing interest [38–40]. For orthopedic joint surgeons, physician ratings by patients were found to be no different between academic *vs.* non-academic surgeons which further questions the reliability of hospital rankings [39]. With all top-ranked USNWR hospitals in the present study being someway affiliated with teaching in some capacity, the survey results from prior studies may require further study. Other studies demonstrated that board certification, being well known for a specific area of expertise, and health insurance in-network providers may be the most important factors influencing patient selection of an orthopedic sports medicine physician [38]. The volume of conflicting findings regarding the quality of care provided by ranked hospitals undermines the ability of the USNWR to accurately capture what patients value most in finding the best surgeon for their orthopedic care [19].

Certain limitations are present in our study for consideration. The data derived from this study included Medicare patients who underwent elective TKA. Thus, only surgeons who treat Medicare-eligible patients were included. There is the possibility of variation in the way each individual procedure was performed. Adjusted complication rates were limited to 30-days postoperatively. Use of an administrative database prevented us from comparing important clinical metrics such as longer-term complications, preoperative function status, short- and long-term functional and radiographic outcomes, and postoperative patient satisfaction. Additionally, complications could have been related to postoperative care or the patient’s own negligence and may not have been accurately considered for this study. The quality of an operation is not only related to the complication rates, but to the postoperative functional recovery of patients, thus complication rates do not fully reflect the skill level of surgeons and should be interpreted in the appropriate context. Moreover, at the consumer level, we were unable to analyze and link specific complications to each surgeon as the publicly available data utilized for this study included only an adjusted complication rate. The ProPublica Surgeon Scorecard reports adjusted complication rates which represent a surrogate for each specific surgeon. There may be inaccuracies with this methodology. However, with the large sample size and consistent mixed-effects methodology employed to each surgeon, our study was primarily focused on the outliers (surgeons with the highest complication rates). Surgeon characteristics also may include some inaccuracies; however, each surgeon was cross-referenced from multiple publicly available sources to ensure accurate age, years in practice, and participation in a fellowship. To minimize bias, we included all surgeons who operated on Medicare-eligible patients at USNWR ranked hospitals during this time if they performed a minimum number of procedures and thus, there were no exclusions. A future study should attempt to compare ranked *vs.* unranked hospitals after controlling for surgical volume, location, and other variables. Despite the limitations, the current study is the first of its kind to report individual-level data of surgeons performing elective TKA at USNWR top-ranked orthopedic hospitals.

## Conclusions

Complication rates of total knee arthroplasty surgeons may be utilized by patients and hospitals to gauge quality of care. Certain surgeon factors including younger age and lower case volume may influence complication rates of surgeons performing TKA at USNWR top ranked orthopedic hospitals.

## Data Availability

All data are publicly available. The Centers for Medicare & Medicaid Services (CMS) from which the data are derived have not verified and are not responsible for the statistical validity of the data analysis or the conclusions derived by the authors.

## Abbreviations

ACR: Adjusted complication rate
CMS: Centers for Medicare and Medicaid Services
ICD-9-CM: International Classification of Diseases, Clinical Modification
NPI: National Provider Identifiers
SAF100: Standard Analytical Files
SPSS: Statistical Package for the Social Sciences
STD: Standard Deviation
TKA: Total knee arthroplasty
USNWR: United States News & World Report
